# The complete chloroplast genome of *Asarum pulchellum* Hemsl. (Aristolochiaceae: *Asarum* L.)

**DOI:** 10.1080/23802359.2021.1992317

**Published:** 2021-10-20

**Authors:** Gang Wang, Xuanjiao Bai, Pei Cao, Jianping Han, Zheng Zhang

**Affiliations:** Institute of Medicinal Plant Development, Chinese Academy of Medical Sciences & Peking Union Medical College, Beijing, China

**Keywords:** *Asarum pulchellum*, medicinal plant, chloroplast genome, phylogeny

## Abstract

*Asarum pulchellum* Hemsley 1890, belonging to Aristolochiaceae family, is a kind of folk herb resource with certain toxicity. In this study, we acquired the complete chloroplast genome of *A. pulchellum* for its accurate species identification and phylogenetic analysis. The genome is 177,905 bp in size with a typical circular quadripartite structure, consisting of a large single-copy region, a small single-copy region and a pair of inverted repeats. In total, 136 genes were annotated, including 92 protein-coding genes, 36 tRNA genes and eight rRNA genes. In the phylogenetic analysis, *A. pulchellum* and *A. sieboldii* formed a sister clade. The chloroplast genome helps further studies of taxonomy and genetic evolution of Aristolochiaceae family.

*Asarum pulchellum* Hemsl., namely Changmao Xixin in Chinese, is a perennial herbaceous plant belonging to *Asarum* genus of Aristolochiaceae family (Kelly 2001). In China, it is mainly distributed in Hubei, Sichuan, Yunnan and Anhui provinces (Lin et al. 1995). *A. pulchellum* contains multiple medicinal components, with the effects of lung warming, dehumidification and pain relief, which can be used to treat cold cough, rheumatic arthralgia, stomachache and other ailments (Kopyt Ko et al. 2013). It is used locally as an endemic herbal resource. However, like other species in *Asarum* genus, *A. pulchellum* also features toxic compounds, e.g., safrole and aristolochic acid, which can cause nephrotoxicity and carcinogenicity when ingested inappropriately (Michl et al. 2017; Han et al. 2019). There are approximately 90 species in *Asarum* genus, whose chemical constituents are divergent (Wu et al. 2021). The misuse of raw materials may adversely affect the safety and effectiveness of their clinical application. So far, there is still a lack of research on *A. pulchellum*, and it is urgent to establish a method for the accurate identification of *A. pulchellum* to ensure its correct use.

This study aims to determine the complete chloroplast genome sequence of *A. pulchellum*. On one hand, it can be considered as a DNA super barcode for species identification of *A. pulchellum* to ensure the accuracy of the original medicinal materials and guarantee the effectiveness and security of its clinical application; on the other hand, it helps gain a better understanding of the genetic and evolutionary relationship of *Asarum* genus.

The specimen of *A. pulchellum* was collected from Wenchuan County, Sichuan Province, China (30°53′40.7″ N, 103°18′37.4″E), and deposited (accession number: YDZW0260) in the Herbarium of Institute of Medicinal Plant Development, Chinese Academy of Medical Sciences (http://www.implad.ac.cn/cn/index.asp, Yulin Lin, linyulin@hotmail.com). The fresh leaves were subjected to cryogenic grinding in liquid nitrogen, then the genomic DNA was extracted using Plant Genomic DNA kit (Tiangen Biotech, Beijing, China) in accordance with the manufacturer’s instructions. The PCR libraries were constructed with 350 bp insert size using the genomic DNA, and 150 bp paired-end reads were sequenced on the Illumina NovaSeq platform. The original sequencing data were assembled into a complete chloroplast genome using NOVOPlasty, which was annotated with CpGAVAS2 (Dierckxsens et al. 2016; Shi et al. 2019).

The complete chloroplast genome of *A. pulchellum* (GenBank accession number: MZ440306) is 177,905 bp in length, and the total GC content is 37.75%. The genome features a typical annular quadripartite structure, including a large single-copy (LSC) region and a small single-copy (SSC) region of 90,885 bp and 8,920 bp respectively, which are separated by a pair of inverted repeats (IRa and IRb) of 39,050 bp. A total of 136 genes were annotated within the chloroplast genome, including 92 protein-coding genes, 36 tRNA genes and eight rRNA genes. Thereinto, 26 genes (15 protein-coding genes, seven tRNAs and four rRNAs) are duplicated in IR regions.

To explore the phylogenetic relationship of *A. pulchellum* with other related taxa, a maximum-likelihood (ML) tree was constructed using IQ-TREE software with TVM + F+R6 nucleotide substitution model (Nguyen et al. 2015). Eighteen complete chloroplast genomes were used in the ML tree (Figure 1), including seven *Asarum* species, eight *Aristolochia* species and three outgroup species (*Ephedra sinica*, *E. intermedia* and *E. equisetina*). The 15 species from Aristolochiaceae clustered together, and the *Asarum* genus and *Aristolochia* genus were separated. *A. pulchellum* and *A. sieboldii* formed a sister clade. Therefore, the complete chloroplast genome sequence of *A. pulchellum* is an essential reference resource for the research of phylogeny and population genetics of Aristolochiaceae family.

**Figure 1.**
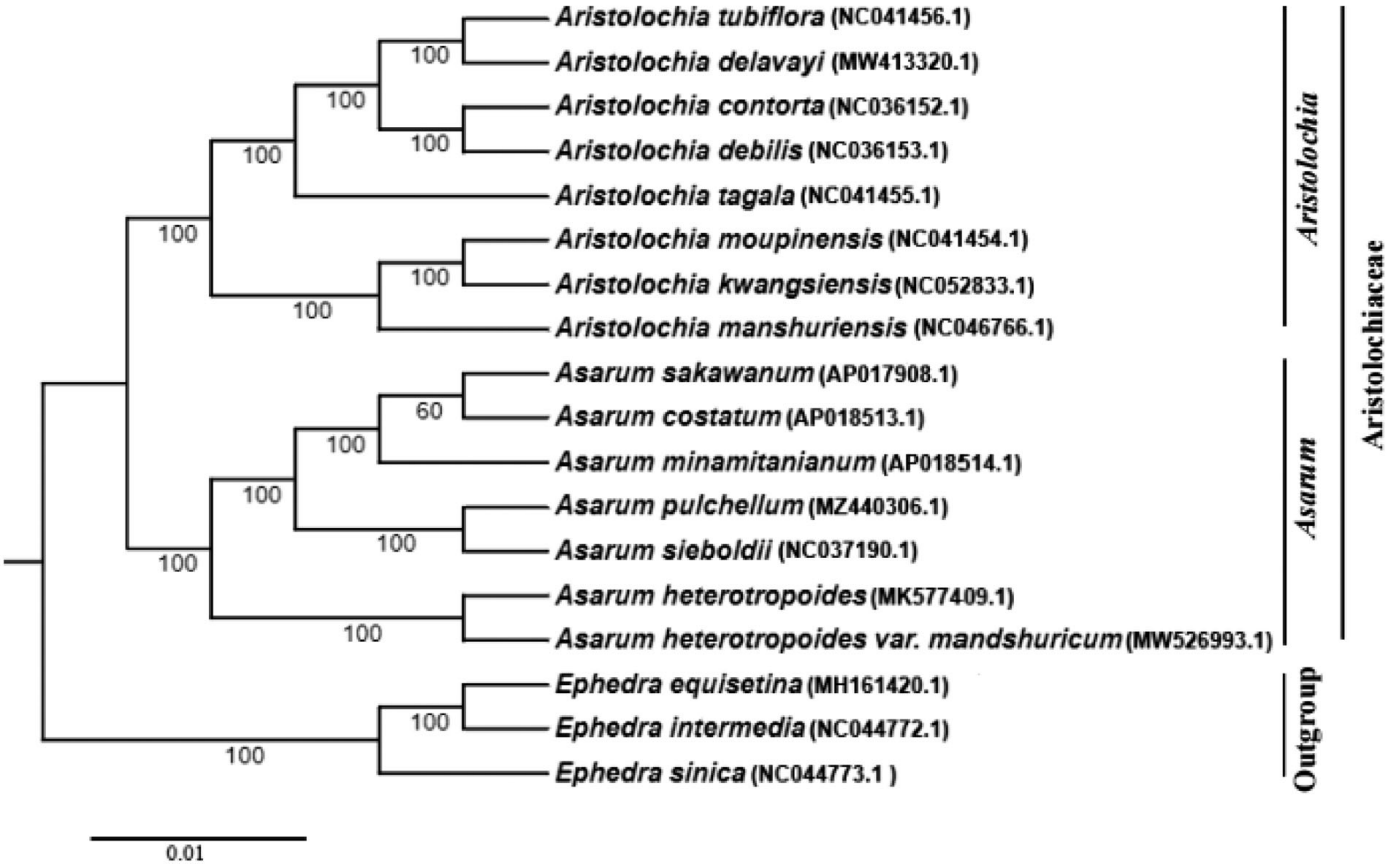
A phylogenetic tree based on 18 complete chloroplast genomes including *A. pulchellum* was constructed with the ML method using TVM + F+R6 model. Numerical value beside each node shows the bootstrap value obtained from 1000 replications. The GenBank accession numbers of genome sequences are shown in the parentheses.

## Data Availability

The genome data that support the findings of this study are available in GenBank of NCBI (https://www.ncbi.nlm.nih.gov/) with the accession NO. MZ440306. The associated BioProject, SRA, and Bio-Sample numbers are PRJNA741848, SRR14949216, and SAMN19909611, respectively.
